# Children’s injury database: development of an injury surveillance system in a pediatric emergency department

**DOI:** 10.1186/s40621-023-00443-8

**Published:** 2023-07-31

**Authors:** Jennifer E. McCain, Ashley E. Bridgmon, William D. King, Kathy Monroe

**Affiliations:** 1 Department of Pediatrics, Division of Pediatric Emergency Medicine, University of Alabama School of Medicine, Birmingham, AL USA; 2 Health Education and Safety Center, Children’s of Alabama, Birmingham, AL USA

**Keywords:** Injury, Injury surveillance, Database, Children, Emergency department

## Abstract

**Background:**

Injuries are the leading cause of death in children and are also a leading cause of all emergency department (ED) visits for children. Obtaining epidemiologic data to define the wide range of childhood injuries for individual communities is challenging. The Children’s Injury Database (CID) is an injury surveillance system developed to collect data from injury-related visits to our tertiary care pediatric emergency department.

**Results:**

During 2021, a total of 15,168 injury visits were analyzed representing 22% of total ED visits (68,834). A total of 2053 injury visits (13.5%) resulted in hospital admission. The 10 leading injury types included: falls, poisonings, motor vehicle collision (MVC), assault, dog bite, burns, sports, pedestrian, bicycle, and all-terrain vehicle (ATV). Admission rates varied by age group with children ages 13 years and older having the highest rate of admission (18.4%). The median length of stay (LOS) for all injured children requiring admission was 2 days while the median LOS for preschoolers was 1 day, the median LOS for school-age children was 2 days, and the median LOS for teenagers was 3 days. While MVCs were the most common cause of vehicle-related injuries, ATV-related injuries had the highest rate of admission (51%).

**Conclusions:**

In this study, teenagers had significantly higher admission rates, lengths of stay, and hospital charges. Black and Hispanic children were under-represented in the number of visits for injuries compared to all ED visits. Further research should focus on disparities in injury-related visits based on race as well as gender. CID has demonstrated that injury surveillance systems can assist with reporting new injury patterns while also acting as a stimulus for new research ideas, planning interventions targeting the most at-risk populations, and evaluating the effectiveness of injury prevention interventions.

**Supplementary Information:**

The online version contains supplementary material available at 10.1186/s40621-023-00443-8.

## Background

Injuries are widely recognized as a significant source of both morbidity and mortality for children. While injuries are the single largest cause of death for children in all age groups over age 1, injuries are also a leading cause of all emergency department (ED) visits for children (Dorney et al. 2020). These ED visits encompass a large range of injuries of varying severity. Most of these injured children seen in EDs will have injuries that can be treated in the ED, and they will be discharged home with various levels of physical and psychological morbidity resulting from the injury (Lee et al. 2022).

The most widely available injury-related statistics are derived from epidemiologic data regarding children who suffer fatal traumatic injuries. While these data certainly describe an important subset of children with the most severe outcomes from their injuries, this shows only a fraction of the range of harm associated with injuries to children. For every child who dies from a traumatic injury, thousands of children sustain injuries from similar mechanisms that require visits to primary care offices, urgent care facilities and EDs where many will require hospitalization. Unfortunately, information describing these children with nonfatal injuries is much more difficult to obtain (Carmichael et al. 2022).

Electronic Health Records (EHRs) are not typically designed to gather data to describe a larger group of individuals with similar injuries. Similarly, insurance records fall short on providing enough detail to describe individual injuries well enough to be able to define the characteristics of the injured individual, the forces of energy causing the injury, the environment in which the injury occurred, or the patterns of injuries resulting from the event.

Identifying, analyzing, and interpreting injury data is an integral step toward effective injury prevention practice at the local, state, national and international levels (Zonfrillo et al. 2014). Establishment and maintenance of injury surveillance databases are invaluable instruments for evaluation research. Local injury surveillance systems effectively identify and describe a large majority of pediatric injuries occurring in a community. Data extracted from these systems can then be used to design public health programs to address injury patterns in order to more appropriately target the most at risk populations with preventative measures (Ferguson et al. 2013).

In an effort to more clearly define the burden injuries cause in our catchment area, our research team designed the Children’s Injury Database (CID). This database is an injury surveillance system that collects information regarding injured children age 0–16 years who are treated (and then are discharged home, are transferred to another facility, or are admitted to the hospital) in an urban free standing tertiary pediatric ED with an annual patient volume of 74,000 patients/year.

## Results

During 2021, our Emergency Department (ED) treated 15,168 injury visits, which accounted for 22% of all ED visits at the hospital.

### Demographics

Males represented a higher proportion of injury visits compared to females (56.8% vs 43.2%, respectively). White children represented the highest proportion of injury visits (56.6%) followed by Black children (41.1%), Hispanic children (0.4%) and others (1.9%). In evaluating racial differences in ED visits for injuries, white children were found to be over-represented among all injury visits when compared to their overall ED visit proportion (56.6% injury vs 40.9% for all ED visits). Black children were under-represented among injury visits compared to all ED visits (41.1% injury vs 50.9% all ED visits) and Hispanic children were greatly under-represented as injury visits compared to all ED visits (0.4% injury vs 6.9% all visits), see Table 1.

**Table 1:** Demographics of pediatric patients presenting to a single center pediatric emergency department in 2021

Total injuries	*n* = 15,168	(22% of all ED visits)
		%
Gender of patients with injury	Males	56.8
	Females	43.2
Racial distribution of patients with injury	White	56.7
	Black	41.1
	Hispanic	0.4
	Other	1.8
Ages of patients with injury	Mean: 7 years	
	Median: 6 years	
	Mode: 1 year	
Season in which injury occurred	Spring	27.6
	Summer	26.2
	Fall	25.3
	Winter	20.9
Disposition of patients with injury	Admission	13.5
	Discharged	79.6
	Other	6.9
Length of stay for patients with injury (mean)	Admissions mean: 5.9 days	
	Discharged mean: 3.1 h	
Length of stay for injury admissions by age group (median)	All injured children: 2 days	
	Preschool: 1 day	
	School-Age: 2 days	
	Teenagers: 3 days	
Insurance status of patients with injury	Private insured	40.0
	Govt./Public Health Ins. Program	56.9
	Self-pay	3.1

The mean age of children presenting to the ED for injury visits was 7.0 years, the median age was 6.0 years, and the mode was 1 year old.

### Seasonality

The mean monthly number of injury visits was 1264; however, April was the leading month for injury visits at 1552 (representing 10.2% of all injury visits for the year). Spring (March, April, May) was the leading season for injury visits and represented 27.5% of all injuries (See Table 1).

### External cause of injury

Our top ten leading external causes of injury include falls, poisonings (unintentional and intentional), MVC (motor vehicle collision), assault, dog bite, burns, sports, pedestrian, bicycle, and ATV (all-terrain vehicle), see Table 2. Among transport-related injuries, ATV visits had the highest proportion of admission (50.9%) followed by motorcycle (36.3%), MVC (22.5%), pedestrian (17.2%) and bicycle (11.8%) (See Table 3).

**Table 2:** Median total charge comparisons of 2021 ED injury visits versus 2021 ED visits (all causes) by injury type (ED injury visit median/ED visit (all causes) median charges) for pediatric encounters at a single center pediatric emergency department for the most common injuries seen

	Observation (N)	Median charge ratio
Falls	4873	1.3
Poison	837	3.7
MVC	820	1.1
Assault	312	1.8
Dog bite	304	0.3
Burns	295	0.5
Sports	293	1.1
Pedestrian	244	1.9
Bicycle	211	1.6
ATV	163	5.6

**Table 3:** Vehicle-related injuries treated at a single center pediatric emergency department in 2021

Vehicle-related injury category	Admitted (*N*)	Total (*N*)	Median LOS (days)	% Admitted
Bicycle	25	211	1.0	11.8
Motorcycle	12	33	2.0	36.3
MVC	184	817	2.0	22.5
Pedestrian	42	244	1.0	17.2
ATV	83	163	2.0	50.9

### Outcomes

The median ESI for all patients evaluated in our ED for an injury-related visit in 2021 was 4.0. Of the patients requiring admission for their injury, the mean ESI given to these patients was 2.6 with the median being 3. Comparing that to our discharged patients, the mean ESI of a patient being discharged was 3.7 while the median ESI for discharged patients was 4. Level 1 or 2 trauma team activations represented 2.7% of total injury visits (410 visits).

The proportion of injury visits resulting in admission was 13.5% while 79.5% were discharged and 7.0% included other outcomes (transfers, AMA, expired, other). The admission rate for all children presenting to our ED in 2021 was 16.5%. When comparing non-injury-related visits to injury visits, there was a significantly (30%) higher proportion of non-injury-related visits requiring admission compared to injury visits, 17% versus 13%, respectively, *z* = 11.7, *p* < 0.00001.

Injury admission rates varied significantly by age group, with teenagers having the highest rate of admission (18.4%), followed by school-age (12.5%), and preschool (12.1%) (See Table 4).

**Table 4:** Age groups for all injury visits seen in a single center pediatric emergency department in 2021

Age group	Admitted		Discharged		Other		Cumulative total
	Frequency (N)	Percent (%)	Frequency (N)	Percent (%)	Frequency (N)	Percent (%)	
0–5 years	833	12.1	5717	83.2	325	4.7	6875
6–12 years	648	12.5	4259	82.1	278	5.4	5185
13 + years	572	18.4	2076	66.8	460	14.8	3108
Total	2053	13.5	12,052	79.5	1063	7.0	15,168

The mean length of stay (LOS) for patients requiring admission was 5.9 days, and the mean LOS for those discharged was 3.1 h. The median LOS for all injured children was 2 days. The median LOS by age group was 1 day for preschool, 2 days for school-age, and 3 days for teenagers. The median LOS for children discharged after injury visits remained constant across age groups at 3 h (See Table 1).

We compared the relative median charges of injury-related ED visits to the 2021 median gross charge for all ED visits. Injury ED visits (all ages) were 33% higher compared to the median gross charge for all visits in 2021. Increased charges for ED injury visits compared to all ED visits differed by age group: preschool children’s charges were 12% higher than charges for all visits, school-age children’s charges were 33% higher than all charges, and teenager’s charges were 80% higher than all charges. Injury ED visit median charges also differed by injury type (see Table 2). Primary insurance categories covering ED injury visits were government/public health insurance programs (56.9%), private insurance companies (40.0%) and self-pay (3.1%) (See Table 1).

## Discussion

Injury prevention specialists search for quality data to demonstrate the epidemiology of injuries in a variety of at-risk populations and communities. Development of the most effective injury prevention interventions is contingent upon detailed surveillance injury data that is difficult to obtain from various sources of generalized health statistics (Stone et al. 1999). Injury surveillance systems have proven effective in the collection of data demonstrating the frequency and severity of injuries, in the recognition of new and recurrent patterns of injury, and in the assessment of successes and limitations of injury prevention programs (Warda 2003).

ED-based injury surveillance systems offer the ability to investigate the gamut of injury in a local urban district ranging from the most minor to the most severe, including deaths (Zuckerbraun et al. 2004). Despite the ease of availability of mortality data, morbidity data more completely describe the range of childhood injuries (King 1991). Collecting data via local injury surveillance systems, such as CID, is likely to give a comprehensive perspective of the scale of the problem of childhood injury in a community (Adirim et al. 1999). For example, studies which use hospital discharge and mortality data would not include many of the more common and milder injury mechanisms, such as falls. Falls are the most common cause of ED visits for children nation-wide and were also found to be the most common cause of ED visits at our institution in 2021. As can be seen from our data in Table 1, most of the children evaluated for injuries were discharged home (79.5%) and therefore would not have been included in an admission or a level 1 trauma database.

Prior studies have shown that ED physicians are not reliably able to collect sufficient details regarding the circumstances surrounding an injury, but when records were reviewed within 24 h for chart abstraction, nearly all needed data points had been included in the record by a medical staff member (Adirim et al. 1999). While we are unable to provide next day review, our team performs routine review of the database and data quality checks to ensure accurate assignment of diagnosis codes. Using an earlier version of the International Classification of Diseases (ICD) system, "E-coded" (used to indicate external cause of injury) hospital discharge databases were found to be a valuable source of information for monitoring occurrences of serious, nonfatal injuries and targeting at-risk groups for prevention interventions (King 1991). This study was based upon the newer version ICD-10 system which provided S, V, Y, X, W and T codes for more accurate and detailed external cause of injury information. We were also able to review a brief history (HPI) for each case.

This project began out of a need for quick access to more detailed injury pattern data for a variety of reasons including targeted prevention, media requests, grant applications, evaluation of on-going prevention activities, and stimulating new research and educational projects in injury prevention. Thus far, we have utilized this database to stimulate further research on ATV-related injuries, firearm-related injuries, self-harm ingestions and a variety of other topics. The database has also proven useful for annual reports and for application for funding for injury prevention efforts.

On review of the data for admissions, when we compared the rates of admissions for each age group, we were quickly able to see the rates of admission (as well as median cost of visit) are significantly higher for adolescents presenting to our ED for injury-related complaints than for younger children presenting for evaluation after an injury (See Table 4).

Our database also allows for identification of high risk but low incidence injuries. For example, we can look at all injuries that occurred last year related to any type of vehicle. As we do that, we find that there were 1816 visits related to injuries associated with a vehicle. This comprises 12% of all injury visits. ATV’s are the fourth most common cause of vehicle-related injuries at our institution, but children with injuries from ATV’s have an extremely high rate of admission at 51% compared to the far more common MVC injury admission rate of 25% (See Table 3).

Our study shows that MVCs are the third leading cause of childhood injury at our institution, similar to national rates (Table 2). Of note, nationally the rates of ATV-related injuries have decreased (https://www.cpsc.gov/s3fs-public/2018AnnualReportofATVRelatedDeathsandInjuries.pdf?VGaf1cuZ_D0SGxct2eRpZUwcgME4LKDy). While ATVs were not the most common motor vehicle injury in this study, the admission rates for these injury types were considerably higher than for other vehicle-related injuries.

As seen in prior studies, males (57% of injury visits) more frequently present for evaluation of injuries than females (Table 1) as compared to our overall ED population that consistently is 52% male (https://www.cdc.gov/injury/features/child-injury/index.html).

The seasonality of injuries seen in Table 1 is not surprising. The rates for winter do not significantly decrease, which is justifiable as this particular study occurred in a southern state with very mild winters leading to more outdoor activities year-round.

As funds for community outreach tend to be difficult to obtain, an ED database such as CID also can easily identify ZIP code areas with highest injury rates of a variety of injury types. Obtaining this data allows for targeted injury prevention to the most at-risk groups.

### Limitations

The limitations of an ED database primarily consist of the lack of accurate complete data. As mentioned above, the team spent additional time reviewing a brief history, but if data were not present in the chart, there was no mechanism for gaining that information. Our team includes two ED physicians who act as liaisons to the other ED providers and share education with them describing why the documentation of details (such as helmet use, type of vehicle, etc.) are important for injury surveillance. Having electronic health records with more comprehensive templates could address this issue in the future.

A second limitation of using a hospital-based ED database such as this for community injury surveillance is that it cannot include every child who is injured in our community. Patients who die in the field will not be included in an ED database such as the CID. However, working with state death review teams and local coroner offices has proven helpful in some of our focused studies to ensure that the most serious of childhood injuries are included in our evaluations. Some patients with minor injuries will not be seen in an ED as they may seek care in their pediatrician’s office, an urgent care facility, a community ED, or the caregivers may choose not to have the injury evaluated by a medical professional. In evaluating the demographics of our injured patients, we found that Black and Hispanic children were under-represented among the total number of injured children. Others have shown that minority groups may be less likely to utilize EDs for a range of medical conditions (Zhang et al. 2019). We have begun to study race and gender disparities in ED injury visits to elucidate the causation of these differences.

## Conclusion

ED injury surveillance can be a valuable tool for understanding the epidemiology of serious, nonfatal forms of child injury. In this study, teenagers were found to have a significantly higher admission rate, length of stay, and hospital charge. Black and Hispanic children were under-represented in the number of visits for injuries compared to all ED visits. Further research should focus on disparities in injury-related visits based on race as well as gender. CID has highlighted local area and state priority injuries for community-based injury prevention programs and can support statewide policy efforts. ZIP codes have been collected and we plan to determine which injuries occur more frequently in which areas to target injury prevention efforts. Hospital web-based messaging, blogs, podcasts and other forms of injury education can be data-driven and location-specific. We have found the use of this local ED database to be beneficial in evaluating trends like the impact of a pandemic on injury patterns as well as for the reporting of new injury patterns, stimulating new research, targeting specific high-risk areas, and for quick access to data.

## Methods

The CID research team included a data analyst, research coordinator for Alabama Safe Kids; an epidemiologist, professor emeritas, a professor of pediatrics and chair of emergency medicine and an emergency medicine pediatrician and medical director of Alabama Safe Kids. All are also members of Injury Free Coalition for Kids. The team applied and received approval at our University and hospital IRB administrations for this project.

In order to retrieve data related only to injury visits, our hospital’s Information Systems analysts searched ED visit primary and secondary diagnoses that fell within the codes provided by the 2020 International Classification of Diseases, 10th Revision, Clinical Modification Injury Diagnosis Framework for Categorizing Injuries by Body Region and Nature of Injury (Hedegaard 2020). Final “Injury Category” assignments were made by applying ICD-10 codes as outlined in the Additional file 1: Appendix—Injury Code Ranges Used in Assigning Injury Categories. Epidemiologic, financial, clinical and outcomes variables were provided by our hospital Information Systems in an Excel format and descriptive data analyses were performed using Epi Info Version 7 (CDC).

Variables collected by CID for each injury visit include: patient name, medical record number, residence ZIP Code, county, city, state, total charges, primary insurance company, ED physician disposition, discharge location, Emergency Severity Index (ESI) score, injury category, age (whole years), race, gender, discharge month, season, length of stay (LOS) in hours for those patients discharged from the ED, LOS in days for those patients admitted to the hospital, total charges, ED diagnosis, primary diagnosis, S Codes, V Codes, Y Codes, X Codes, W Codes, T Codes, and information in the History of Present Illness (HPI) section of the medical record. All diagnosis codes were provided using the ICD-10 categorization scheme for injury-related ED visits. An Emergency Severity Index (ESI) score is assigned to every patient upon triage completion by our nursing staff and correlates to the level of acuity of each patient. An ESI score of 1 is the highest level of acuity while a score of 5 is the lowest acuity level. In order to compare outcomes at different ages, age groups were assigned as follows: preschool (0–5 years), school-age (6–12 years), and teenagers (13 + years).

All injury visits identified were included in this study. When data variables were missing from individual visits in the database, members of the core research team reviewed data included in the HPI section of the note. If missing data was available, it was added to the database in the appropriate location. Data quality checks were routinely provided on random samples of specific injury categories by comparing injury category data to HPI text entries. When a patient was seen for a repeat encounter, each visit was included into the database as a visit.

Basic descriptive analyses were performed using Epi Info Version 7 (Centers for Disease Control and Prevention (CDC)), which included frequency analyses of categorical data and mean and median analyses of numerical data. Z test of proportions was used to compare non-injury-related visits to injury visits. Cross-tabulations of categorical data were provided as needed.

### Supplementary Information

**Additional file 1:** Injury code ranges used in assigning injury categories.

## Data Availability

The datasets used and/or analyzed during the current study are available from the corresponding author on reasonable request. These cannot be publicly shared as they have been pulled from a hospital HER and include protected health information. Our research team would share data within the requirements, standards and protections of our institutional IRB approval for this research.

## Abbreviations

ED: Emergency department
EHR: Electronic health records
CID: Children’s injury database
IFCK: Injury-free coalition for kids
ESI: Emergency severity index
LOS: Length of stay
HPI: History of present illness
ICD-10: International classification of diseases 10
MVC: Motor vehicle collision
ATV: All-terrain vehicle
CDC: Centers for disease control and prevention
